# Nanosecond Laser Cleaning of 10CrNi2Mo3Cu2V Steel: Surface Cleaning, Oxidation Control and Welding Performance

**DOI:** 10.3390/ma19183889

**Published:** 2026-09-12

**Authors:** Donghe Zhang, Yinghao Guo, Xinhui Xu, Yang Chen, Shukai Hu, Zexuan Han, Lijun Yang, Debin Shan, Jie Xu, Bin Guo

**Affiliations:** 1 School of Mechatronics Engineering, Harbin Institute of Technology, Harbin 150080, China; zhangdonghe@hit.edu.cn (D.Z.); yljtj@hit.edu.cn (L.Y.); 2 Zhengzhou Advanced Research Institute of Harbin Institute of Technology, Zhengzhou 450001, China; 3 Key Laboratory of Micro-Systems and Micro-Structures Manufacturing of Ministry of Education, Harbin Institute of Technology, Harbin 150080, China; 4 School of Materials Science and Engineering, Harbin Institute of Technology, Harbin 150080, China

**Keywords:** 10CrNi2Mo3Cu2V steel, laser cleaning, oxide layer, pre-weld treatment

## Abstract

**Highlights:**

• An argon atmosphere is more effective at suppressing secondary oxidation reactions during the laser-cleaning process.
• At an energy density of 7.64 J/cm^2^, the oxygen content on the steel surface decreased to 2.11 wt.% after laser cleaning, and the surface roughness was 5.2 μm.
• Laser cleaning prior to welding improves welding performance.
• Laser cleaning is a promising contact-free method for pre-welding surface preparation.
• The optimal cleaning process parameters for 10CrNi2Mo3Cu2V steel were determined.

**Abstract:**

High-strength steels often require surface pretreatment before welding to remove contaminants and ensure weld quality. Compared with conventional mechanical grinding, which is time-consuming, labor-intensive, environmentally unfriendly, and potentially damaging to the substrate, laser cleaning offers a contact-free alternative to conventional surface-treatment methods and may reduce the use of abrasive or chemical cleaning agents. In this study, nanosecond laser cleaning of oxide films on 10CrNi2Mo3Cu2V steel was investigated in air and argon to determine the optimal process window. A 1064 nm, 100 ns pulsed fiber laser was employed for single-pass scanning at fluences of 5.10–10.19 J/cm^2^. The cleaned surfaces were characterized by SEM, EDS, XPS, laser confocal microscopy, microhardness testing, and vacuum electron-beam welding. In argon, the optimal fluence was 7.64 J/cm^2^, at which the oxide-related surface products were effectively removed in the analyzed area, the oxygen content decreased to 2.11 wt.%, the roughness reached 5.2 μm, and the hardness became comparable to that of the ground sample. At higher fluences, secondary oxidation increased and surface quality deteriorated. The optimized laser-cleaned joints were pore-free and achieved a tensile strength of 881.4 MPa, exceeding that of both the untreated and ground controls.

## 1. Introduction

In recent years, 10CrNi2Mo3Cu2V, a high-strength alloy structural steel, has been increasingly used in aerospace and marine applications because of its heat and wear resistance, and is mainly employed in components such as aviation gears and shafts. However, steel surfaces are prone to contamination. For example, steel surfaces readily oxidize [1]; oxide scale can form during rolling of steel plates and reduce plate quality [2]; and corrosion and rusting occur easily in hot and humid environments [3]. All of these factors shorten the service life of the material. They also affect subsequent processing [4], for example by generating numerous pores during welding and thereby reducing weld quality. Consequently, preweld treatment has become a critical step for ensuring weld quality [5].

10CrNi2Mo3Cu2V is a Cr-Ni-Mo-Cu-V alloy structural steel whose alloying design provides a combination of high strength, heat resistance, and wear resistance. These characteristics support its use in high-load components such as aviation gears and shafts, for which surface integrity and reliable joining are important to service performance. The alloying chemistry and the presence of a rust or oxide layer also make it necessary to establish a material-specific cleaning process window. In particular, the laser parameters must be sufficient to remove the oxide film and surface contaminants while avoiding excessive substrate melting, secondary oxidation, and surface damage. Therefore, cleaning conditions developed for other steels cannot be directly transferred to 10CrNi2Mo3Cu2V without experimental verification.

In this study, the term “surface oxide/rust layer” is used as a general description of the initial surface products, including relatively adherent iron oxides and loosely attached atmospheric corrosion products. The term “oxide scale” is reserved for oxide layers formed during elevated-temperature processing, whereas “rust” specifically refers to corrosion products generated during atmospheric exposure. When the formation history or phase composition cannot be determined conclusively, the general term “surface oxide layer” is used.

Conventional cleaning methods mainly include mechanical grinding, high-pressure water jet cleaning [6], and chemical cleaning. Mechanical grinding is widely used in production because of its simplicity, but it can severely damage the substrate surface and produce nonuniform cleaning, thereby reducing surface quality [7]. High-pressure water jet cleaning has a short cycle but consumes large amounts of water [8], which runs counter to contemporary environmental awareness. Chemical cleaning can selectively remove surface contaminants, but it may harm human health during processing and cause irreversible environmental pollution [7,9]. Therefore, a more efficient and potentially environmentally benign cleaning method is desirable for the high-quality treatment of structural steels [10].

Compared with conventional cleaning, laser cleaning has irreplaceable advantages. First, the laser beam does not contact the workpiece during cleaning [9], so substrate damage is minimal [11]. The laser beam delivers concentrated energy and allows a controllable working range [12], which, when combined with a robotic arm, enables highly efficient operation. When the laser acts on the material surface, contaminants are typically detached as micro- or nanoscale particles, truly enabling green and pollution-free removal. For this reason, laser cleaning is regarded as one of the most promising green cleaning technologies of the twenty-first century [13,14] and is now widely used in aerospace, automotive manufacturing, shipbuilding, and related fields [15].

Over the past few decades, laser cleaning has continued to advance, and a growing body of research has emerged, forming an active research landscape. Studies on laser cleaning of steel surfaces at home and abroad have moved from asking whether rust layers can be removed to asking whether surface integrity, corrosion resistance, and downstream manufacturability can be simultaneously maintained. Guo [16] pointed out that a unified understanding is still lacking regarding corrosion evolution in steel bridges, rust-removal methods, and their structural and environmental effects, while laser cleaning is considered an important alternative because of its high efficiency, low pollution, and contact-free nature. Koh [17] further compared laser cleaning with mechanical cleaning and demonstrated its clear advantages in treating corroded steel surfaces. At the mechanistic level, Zhou [18] categorized laser cleaning into thermal ablation, thermal stress, and plasma shock-wave mechanisms, providing a theoretical framework for subsequent parameter optimization and mechanism analysis.

For specific steels, international studies have focused early on surface treatment of shipbuilding steel and rusted steel. Lu [19] proposed an ultraviolet nanosecond laser rust-removal method for AH36 steel and showed that a fluence of 3.17 J/cm^2^ yielded the best match between surface roughness and corrosion resistance, with corrosion resistance about five times that of the original rusted surface. Tao [20] further examined the effects of power, repetition frequency, and overlap ratio on roughness, microstructure, hardness, mechanical properties, and corrosion resistance in AH36 steel, and found that 40 W, 110 kHz, and 50% overlap produced the best cleaning result, with overall surface performance superior to that of the substrate. Wang [21,22,23] carried out continuous-wave laser-cleaning studies on severely corroded steel components and weathering steel, demonstrating that laser treatment can effectively remove corrosion products and salts and, through process optimization, improve surface cleanliness and delay re-corrosion.

Domestic studies have placed greater emphasis on high-strength steels, engineering process windows, and service adaptability after cleaning. Ma [24] systematically analyzed the effects of fluence and overlap ratio on surface roughness, microhardness, and corrosion resistance in Q345 steel, noting that a moderate increase in energy and overlap can improve surface integrity, whereas excessive energy leads to increased roughness, pits, oxidation, and performance degradation. He [25] identified an optimal window of 4.26 J/cm^2^ and 75% overlap for 20 steel, showing that rust layers can be removed while corrosion resistance is improved. Wang [26] applied response surface methodology and NSGA-II to the Q390 rust layer, indicating that domestic research has evolved from single-factor experiments toward parameter optimization and engineering decision-making. At the same time, the coupling of laser cleaning and welding has become an important direction. Zhu [27] showed that laser-cleaning pretreatment can effectively remove rust and oil from HSLA steel surfaces, create microgrooved textures favorable for laser absorption, and significantly reduce weld porosity and cracking while improving tensile strength and elongation. Seo [5] also demonstrated that laser cleaning can remove by-products after welding, indicating that the technique is applicable not only before welding but also after welding. Turner [28] effectively removed the oxide film from Ti-Al alloy surfaces using argon-assisted cleaning; no defects were observed in subsequent welds, showing that argon can serve as a suitable shielding gas during laser cleaning compared with the atmospheric environment. However, for 10CrNi2Mo3Cu2V and similar high-strength structural steels, a systematic study that integrates macroscopic and microscopic characterization, performance evaluation, cleaning mechanism analysis, and welding response is still lacking.

Although laser cleaning has been investigated for several other steels, the available literature does not establish a systematic, material-specific process window for 10CrNi2Mo3Cu2V or clarify how the cleaned surface affects the subsequent welding response of this alloy. This knowledge gap is important because the cleaning threshold, the balance between oxide removal and substrate remelting, and the susceptibility to secondary oxidation may depend on the alloy chemistry and the initial surface condition. The novelty of the present work is therefore to combine air and argon atmosphere comparisons with surface composition, morphology, roughness, microstructure, hardness, high-speed imaging, and vacuum electron-beam welding evaluation for 10CrNi2Mo3Cu2V steel. This integrated approach links the laser-cleaning process window to the final weld quality rather than assessing surface cleanliness alone.

Accordingly, this study aims to clarify the material-specific laser-cleaning behavior of 10CrNi2Mo3Cu2V steel and its influence on subsequent welding performance. Particular attention is given to the role of the processing atmosphere in suppressing secondary oxidation and to the determination of a laser-fluence range that enables effective oxide-film removal while minimizing substrate remelting and surface deterioration. The evolution of the oxide-removal mechanism with increasing laser fluence is also analyzed, with emphasis on the contributions of plasma shock, particle detachment, and laser ablation. In addition, the effects of optimized laser cleaning on surface composition, morphology, roughness, microstructure, hardness, weld pore formation, and tensile properties are evaluated in comparison with conventional mechanical grinding. Through these analyses, this work establishes the relationship between laser-cleaning parameters, surface oxidation control, and the welding performance of 10CrNi2Mo3Cu2V steel.

## 2. Materials and Methods

### 2.1. Materials

**Figure 1:**
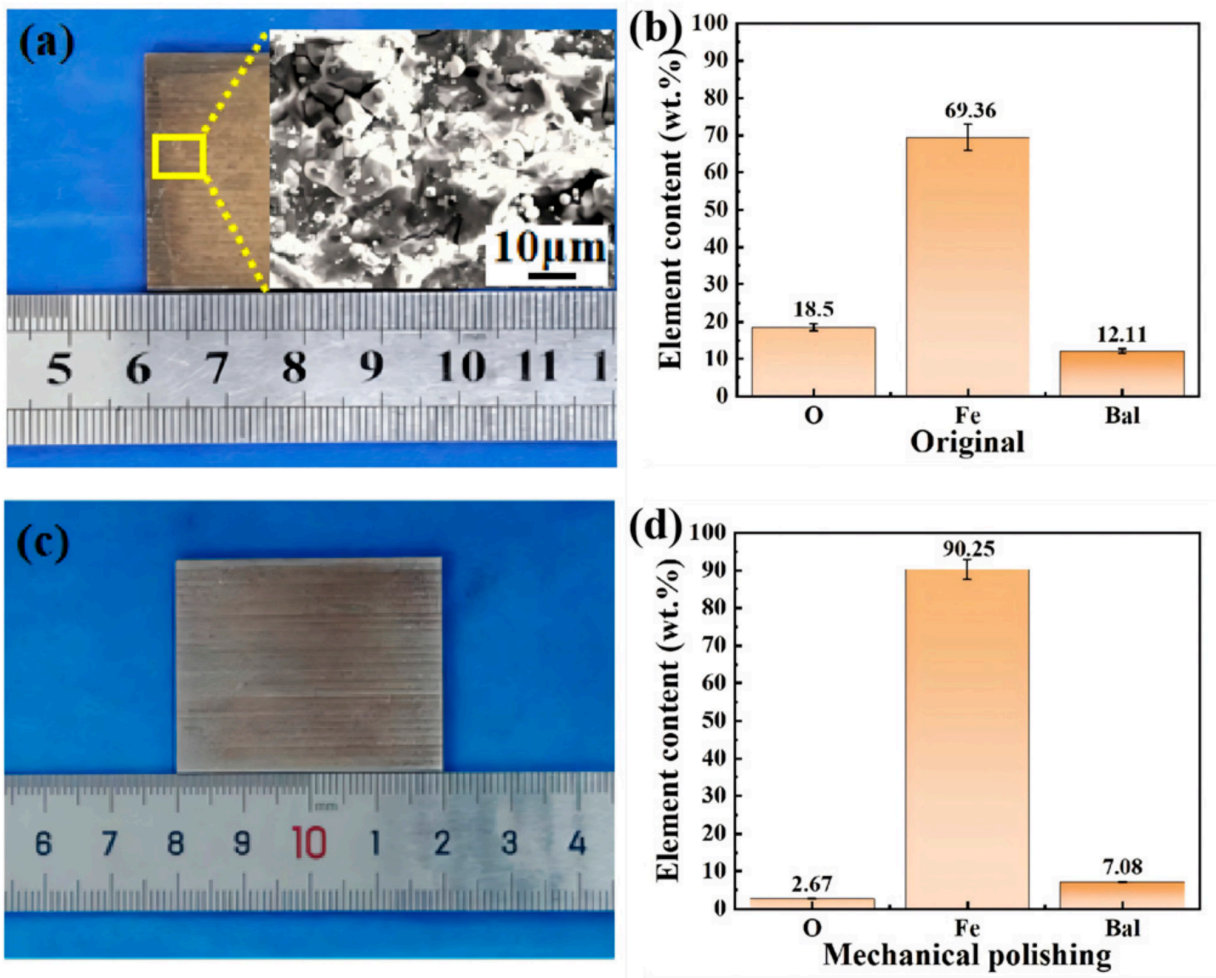
Surface morphology and compositional analysis of the original and ground specimens. (**a**) Macroscopic surface and microstructure of the as-received specimen; (**b**) Percentage composition of elements on the surface of the as-received specimen; (**c**) Macroscopic surface and microstructure of the ground specimen; (**d**) Percentage composition of elements on the surface of the ground specimen.

In this study, heat-treated 10CrNi2Mo3Cu2V steel bars were used, and the specific composition is listed in Table 1. For testing convenience, the bars were wire-cut into specimens measuring 30 mm × 40 mm × 2 mm. The original sample surface was covered by a heterogeneous surface oxide/rust layer consisting of a relatively adherent oxide film and loosely attached corrosion products; the surface morphology and elemental composition are shown in Figure 1a,b, with O content of 18.50 wt.% and Fe content of 69.39 wt.%. The mechanically ground samples were treated with 400-grit sandpaper; after grinding, the oxide layer was essentially removed, and the surface morphology and elemental distribution are shown in Figure 1c,d, with O content of 2.67 wt.% and Fe content of 90.25 wt.%.

### 2.2. Laser Cleaning

The laser-cleaning system mainly consisted of a laser head, laser source, robotic arm, control system, and water chiller. During cleaning, the laser source generated the beam, which was delivered through an optical fiber to the cleaning head, collimated by an internal beam-shaping system, and then scanned by a two-dimensional galvanometer before passing through a flat-field focusing lens to reach the sample surface for bow-shaped scanning. A nanosecond pulsed fiber laser (YLPN-100-30 × 100-1000, IPG, Marlborough, MA, USA) was used in the experiment, and the process parameters are listed in Table 2. Average power, repetition frequency, and scanning speed are the main factors affecting laser-cleaning performance: increasing the power improves the cleaning efficiency, whereas increasing the repetition frequency increases the number of spots per unit area and leads to greater thermal accumulation. Laser fluence (*F*), which couples power, frequency, and spot diameter, is an important variable and can be expressed as Equation (1) [29]:

(1) $$F\;=\;\frac{P_{v}}{f\cdot \pi R^{2}}$$

where *P_v_* is the laser power, *f* is the repetition frequency, and *R* is the radius of a single spot.

The spot-overlap ratio is divided into a transverse overlap ratio *U_x_* and a longitudinal overlap ratio *U_y_*, as given in Equations (2) and (3) [30].

(2) $$U_{x}=\left(1-\frac{v}{\mathrm{Df}}\right)\;\times \;100\%$$

(3) $$U_{y}=\left(1-\frac{L}{D}\right)\;\times \;100\%$$

where *U_x_* is the transverse spot-overlap ratio, *U_y_* is the longitudinal spot-overlap ratio, v is the laser scanning speed, f is the pulse repetition frequency, *D* is the laser spot diameter, and *L* is the displacement between adjacent scan lines perpendicular to the scanning direction. A larger overlap ratio indicates that a greater area is repeatedly irradiated during laser cleaning. In this study, *U_x_* was set to 50% and *U_y_* was set to 90%.

**Figure 2:**
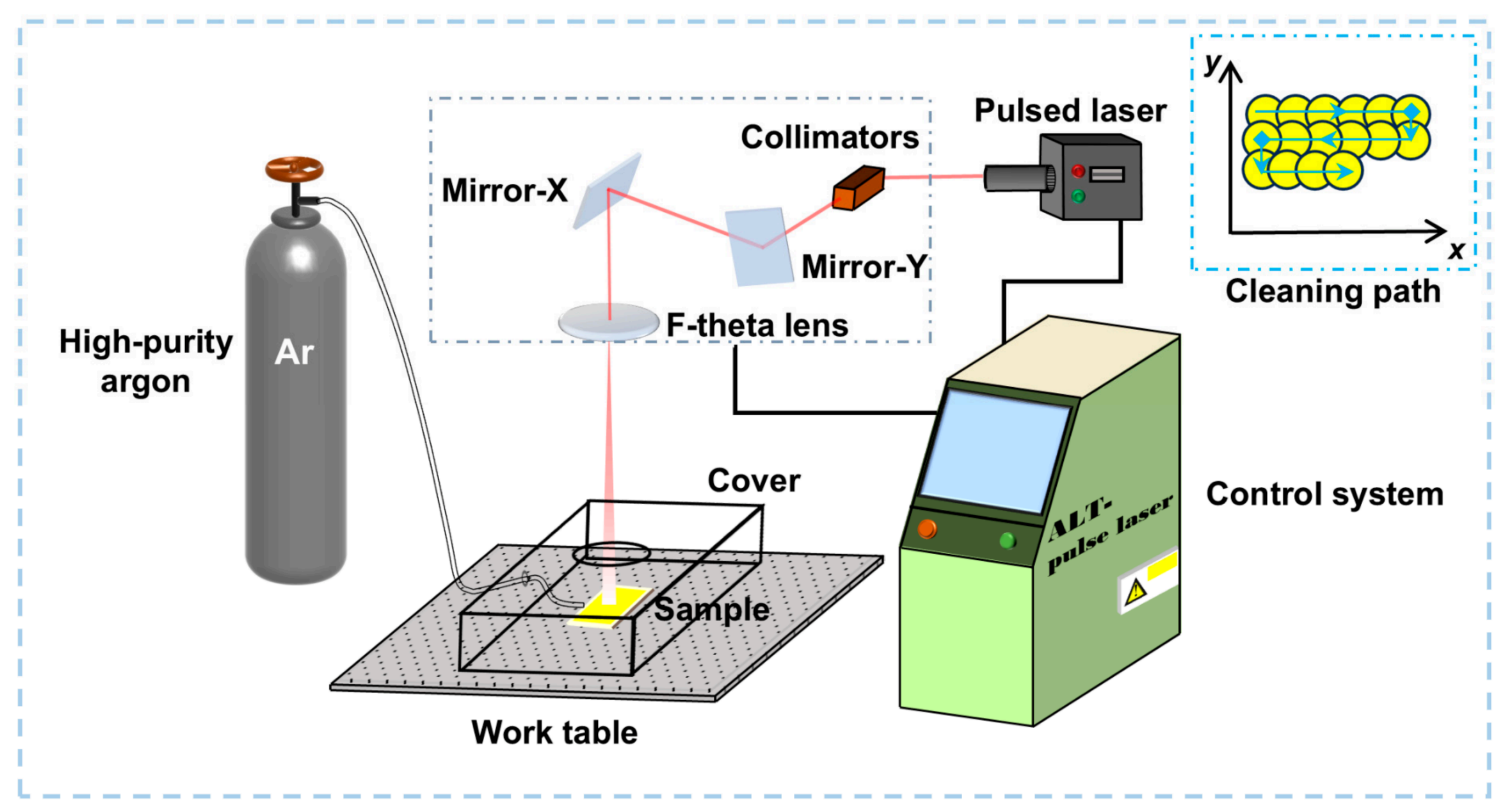
Schematic of the laser-cleaning process.

As shown in Figure 2, the cleaning experiments were divided into two groups. For the argon-protected group, the specimens were placed in a sealed transparent chamber. Industrial high-purity argon was introduced at a flow rate of 10 L/min before and during laser cleaning to reduce the exposure of the heated surface to atmospheric oxygen. The argon purity was 99.999% according to the gas specification. The first group was cleaned with a single scan in ambient atmosphere; the second group was purged with industrial high-purity argon inside a sealed transparent chamber at a flow rate of 10 L/min to prevent oxidation [30]. The process parameters were identical for both groups.

### 2.3. Welding Procedure

**Figure 3:**
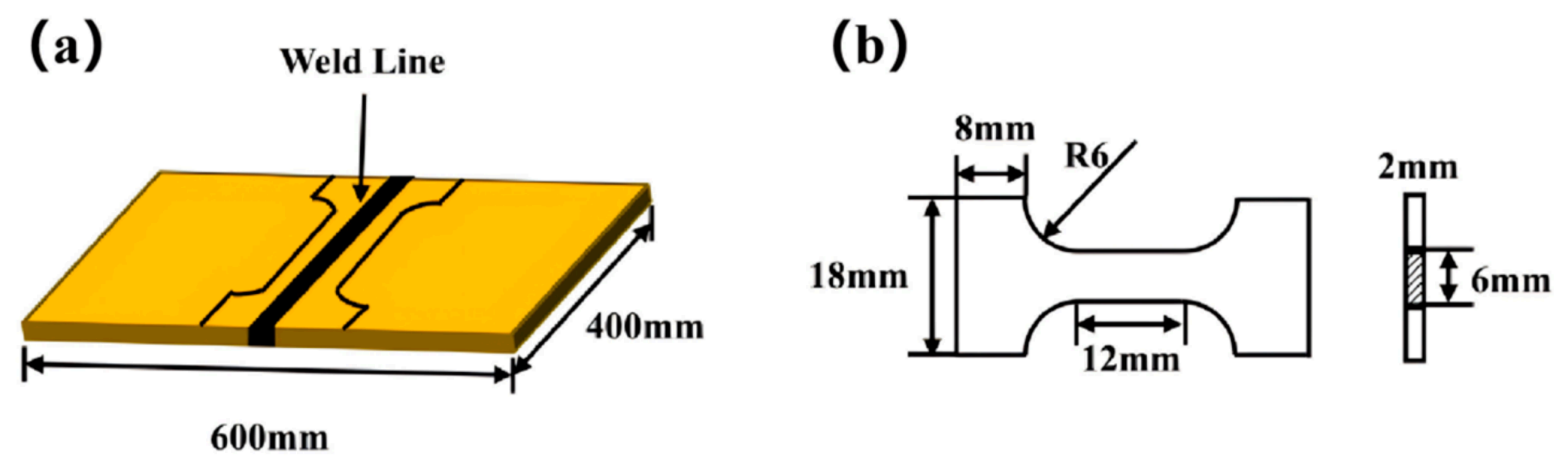
Schematic of the specimen design and dimensions: (**a**) welded specimen; (**b**) tensile specimen.

Tensile specimens were cut from welded samples. Before welding, the weld faces and adjacent side surfaces were ground or cleaned. Vacuum electron-beam welding was then performed with a welding voltage of 130 kV, a welding speed of 15 mm/s, a beam current of 8 mA, and a focus current of 1580 mA. The dimensions of the welded specimen are shown in Figure 3a.

After welding, the welded specimens were cut and machined into tensile specimens for subsequent mechanical testing and weld microstructure observation. The tensile-test dimensions are shown in Figure 3b. For each surface-treatment condition, three parallel welded tensile specimens were prepared and tested, including the untreated, mechanically ground, and laser-cleaned conditions. Three welded tensile specimens were prepared and tested for each surface-treatment condition, including the untreated, mechanically ground, and laser-cleaned conditions. The tensile-strength data were calculated as the arithmetic mean of the three valid measurements, and the dispersion was expressed as the sample standard deviation.

### 2.4. Characterization Methods

The surface microstructure of the specimens before and after cleaning and the internal weld microstructure were observed using an electron probe scanning microscope (JEOL, Tokyo, Japan). The O and Fe contents on the sample surface were measured with the attached energy-dispersive spectrometer (EDS).

XPS measurements were performed using an ESCALAB 250Xi spectrometer (ESCALAB 250Xi, Thermo Fisher, Waltham, MA, USA) equipped with a monochromatic Al Kα X-ray source (1486.6 eV). The binding-energy scale was calibrated by referencing the C 1s peak of adventitious carbon to 284.8 eV. The specimens were electrically grounded during measurement, and charge compensation was performed using a low-energy electron neutralizer. Survey spectra were acquired using a pass energy of 100 eV, an energy step of 1.0 eV, and 5 scans. High-resolution Fe 2p and O 1s spectra were acquired using a pass energy of 20 eV, an energy step of 0.1 eV, and 10 scans. The analysis area was approximately 500 μm in diameter, and the chamber pressure was approximately 1.0 × 10^−7^ mbar.

The XPS data were processed using Thermo Avantage software (5.9). After Shirley background subtraction, the Fe 2p and O 1s spectra were fitted using Voigt-type peak profiles. The same fitting procedure and constraints were applied to all specimens. The Fe 2p fitting considered the spin–orbit components and the multiplet and satellite structures associated with Fe^0^, Fe^2+^, and Fe^3+^ species. The O 1s spectra were evaluated together with the Fe 2p spectra and the EDS oxygen-content results.

Three-dimensional surface morphology and roughness changes were analyzed using a laser confocal scanning microscope (LSCM, OLS4100, OLYMPUS, Hachioji, Japan). Microhardness was measured using a Vickers hardness tester (HUAYIN, Laizhou, China), and the tensile properties of the welded joints were tested with a universal testing machine.

EBSD analysis was conducted on the cross-sections of the specimens to characterize the grain-orientation distribution, grain morphology, and local crystallographic misorientation before and after laser cleaning. The EBSD maps were acquired from cross-sectional regions measuring 57.5 μm × 42 μm for each condition, and the same acquisition and data-processing procedures were applied to all maps. The KAM maps were used to evaluate local orientation gradients, lattice distortion, and strain heterogeneity.

For each experimental condition, three parallel measurements were performed for EDS, XPS, LSCM, microhardness, and EBSD analyses. The same experimental and data-processing procedures were applied to all parallel measurements. The reported values were calculated as the arithmetic mean of the three valid measurements, and the error bars represent the standard deviation.

## 3. Results and Discussion

### 3.1. Cleaning Performance

#### 3.1.1. Macroscopic Surface Morphology

**Figure 4:**
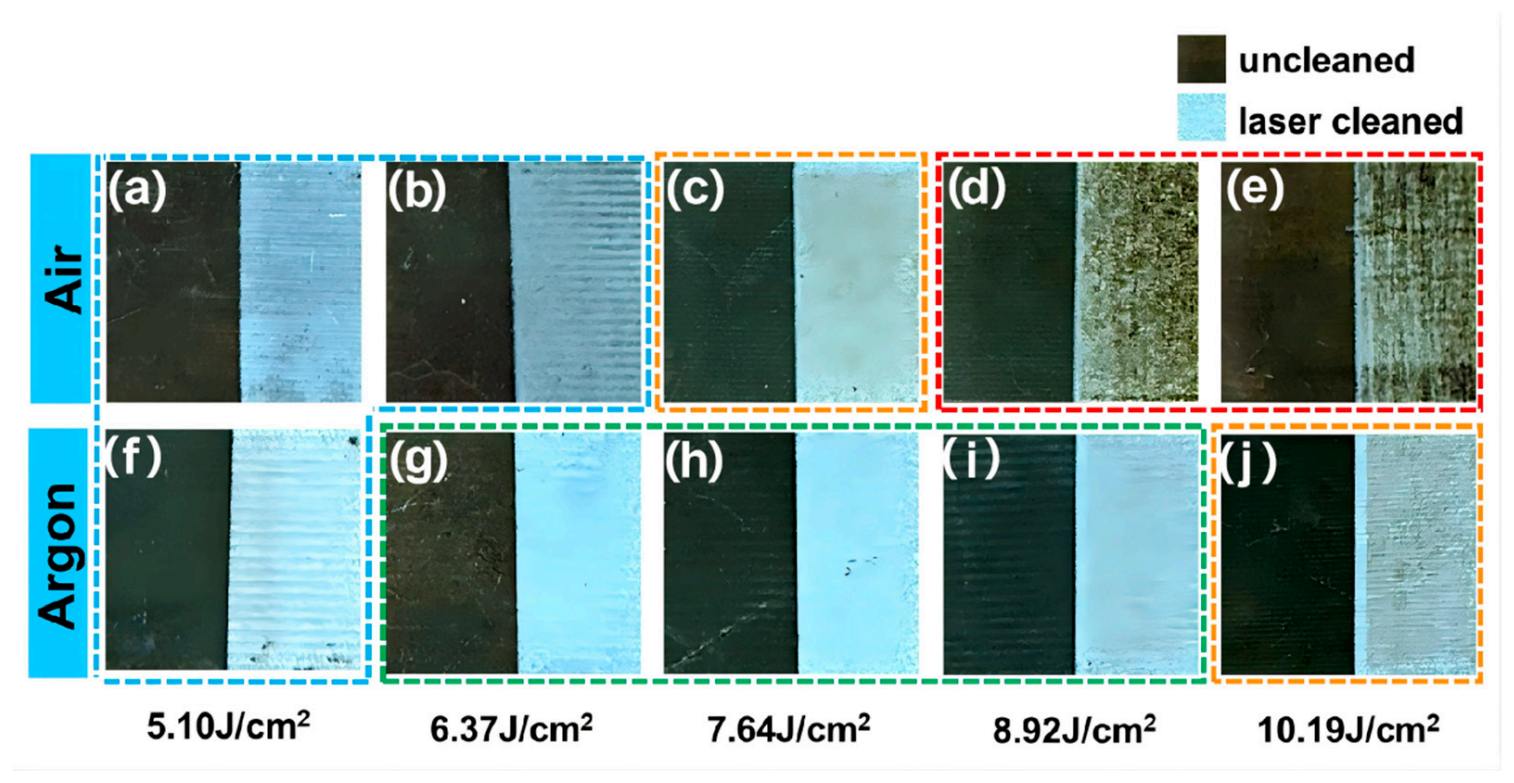
Comparison of specimen surfaces before and after cleaning—(**a**,**f**): 5.10 J/cm^2^; (**b**,**g**): 6.37 J/cm^2^; (**c**,**h**): 7.64 J/cm^2^; (**d**,**i**): 8.92 J/cm^2^; (**e**,**j**): 10.19 J/cm^2^.

Figure 4 shows the macroscopic surface morphology before and after laser cleaning. Figure 4a–e and Figure 4f–j correspond to specimens cleaned in air and argon, respectively. In air, the surfaces remained gray at the lower fluences of 5.10 and 6.37 J/cm^2^. At 7.64 J/cm^2^, the surface became light yellow, while darker brown regions appeared at 8.92 and 10.19 J/cm^2^. In argon, the cleaned surfaces exhibited a brighter metallic appearance at fluences from 5.10 to 8.92 J/cm^2^. At 10.19 J/cm^2^, the surface showed a slight yellow coloration. These changes in color and surface luster indicate differences in the final surface state under different fluences and atmospheres. The corresponding changes in surface composition and chemical state are analyzed in Section 3.1.2 and Section 3.1.3.

#### 3.1.2. Microstructure and Elemental Content

**Figure 5:**
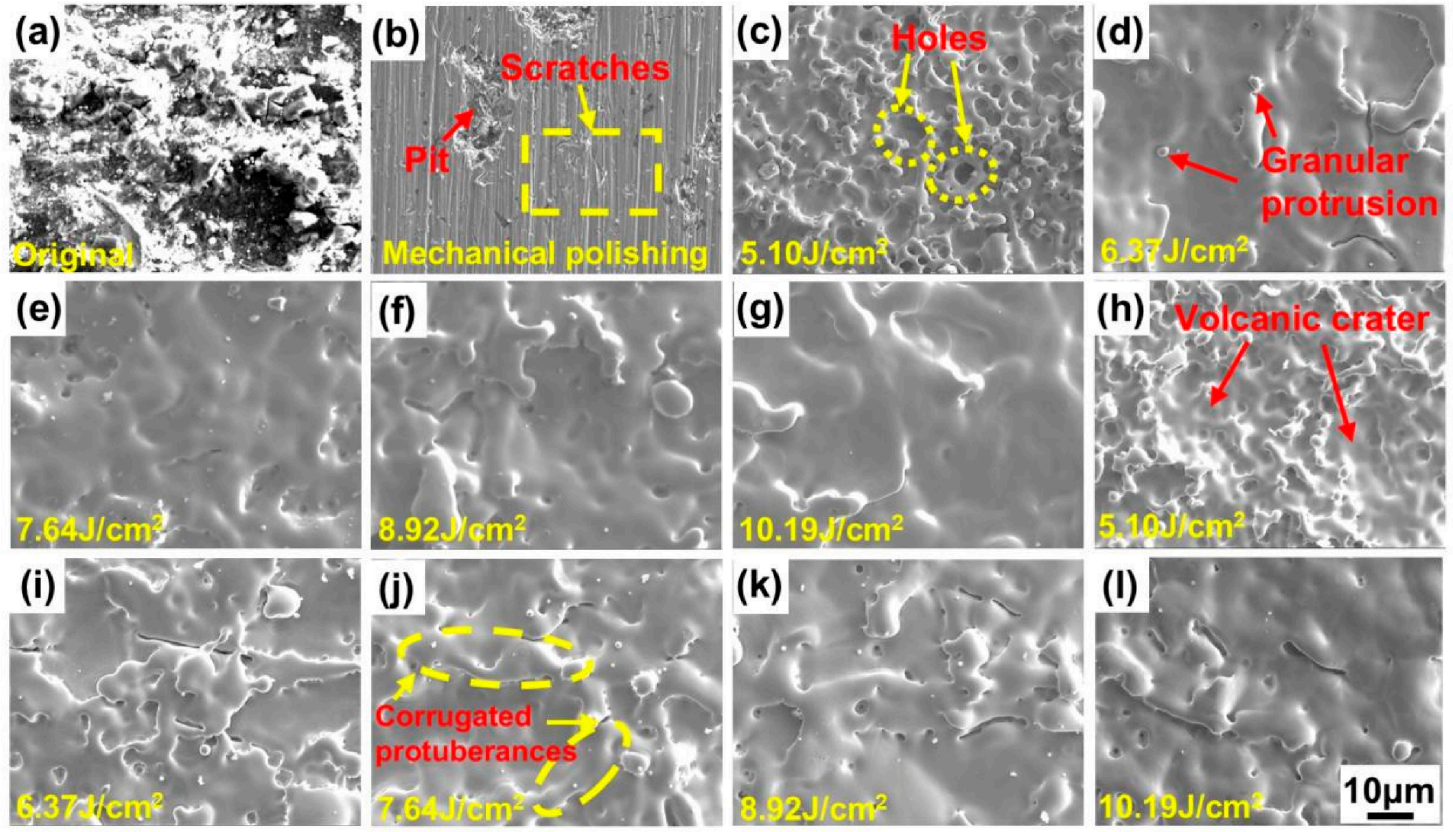
Surface micro-morphology of the specimens: (**a**), original specimen; (**b**), mechanical grinding; (**c**–**g**), air atmosphere; (**h**–**l**), argon atmosphere (×500).

Figure 5 shows the microscopic surface morphology of the specimens. The original specimen surface (Figure 5a) was covered with impurities and attached with numerous contaminant particles. The ground specimen surface (Figure 5b) was covered with scratches, and friction of metal particles formed pits on the surface. After laser cleaning, the surface morphology changed similarly in both atmospheres. At 5.10 J/cm^2^, the energy input was insufficient and only part of the oxide film was removed; some local regions melted under laser irradiation and released gas. During rapid solidification of the melt pool, gas was easily trapped, forming numerous pores and a volcano-crater-like structure (Figure 5c,h). When the fluence increased to 6.37–10.19 J/cm^2^, the surface gradually developed a wavy morphology. This is because the temperature in the laser-affected zone was high enough to melt the surface metal, which then flowed and re-spread, while the surrounding region remained cooler and solidified more quickly, producing the observed morphology due to differences in cooling rate. With further increases in fluence, the molten metal became more fluid, filling surface defects and removing some contaminants, and the surface gradually became flatter with reduced relief.

**Figure 6:**
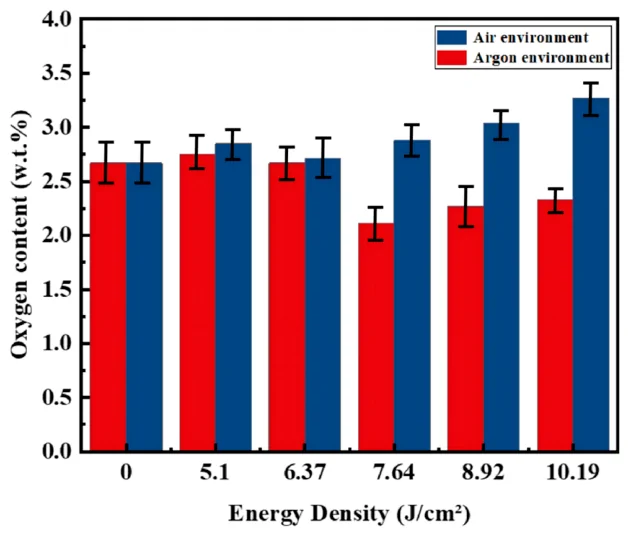
O element content on the sample surface.

Figure 6 shows the variation in O content on the sample surface after laser cleaning of the ground specimens in air and argon. The O content on the original specimen surface was 18.50 wt.% (Figure 1b), whereas that on the ground specimen surface was 2.67 wt.% (Figure 1d). Compared with the untreated specimen, the O content decreased markedly, indicating that mechanical grinding removed most of the rust and oxide layer on the material surface. In air, as the fluence increased from 5.10 to 10.19 J/cm^2^, the surface O content increased gradually from 2.85 wt.% to 3.27 wt.%. Although still far below that of the original surface, it remained higher than that of the ground specimen, indicating that laser cleaning can induce some degree of reoxidation while removing the residual oxide layer. This is mainly because laser irradiation causes the surface to heat up instantaneously. After the original oxide film is removed, a highly reactive fresh metal surface is exposed, and in air this surface rapidly reacts with O_2_ and H_2_O to form a new thin oxide film. In argon, as the fluence increased from 5.10 to 10.19 J/cm^2^, the O content decreased from 2.75 wt.% to 2.11 wt.% at 7.64 J/cm^2^, indicating progressive oxide removal. At 8.92 and 10.19 J/cm^2^, the O content increased slightly to 2.27 wt.% and 2.33 wt.%, respectively. The minimum O content occurred at 7.64 J/cm^2^. Together with the XPS and morphology results, this finding indicates that the oxide-related surface products were effectively reduced in the analyzed area, and that the surface condition was improved compared with conventional grinding. This indicates that the inert atmosphere effectively suppresses reoxidation during laser cleaning, although slight oxidation can still occur at higher fluence because of local high temperatures and trace residual oxygen.

The microscopic morphology changed progressively with increasing laser fluence. At 5.10 J/cm^2^, the surface contained numerous pores and crater-like features, whereas wavy and relatively smooth morphologies developed at higher fluences. These features reflect differences in the surface morphology after laser irradiation. Their relationship with oxide removal, surface melting, and secondary oxidation is further examined by combining the EDS and XPS results with the high-speed imaging observations in Section 3.2.

#### 3.1.3. Elemental Composition and Chemical State Analysis

**Figure 7:**
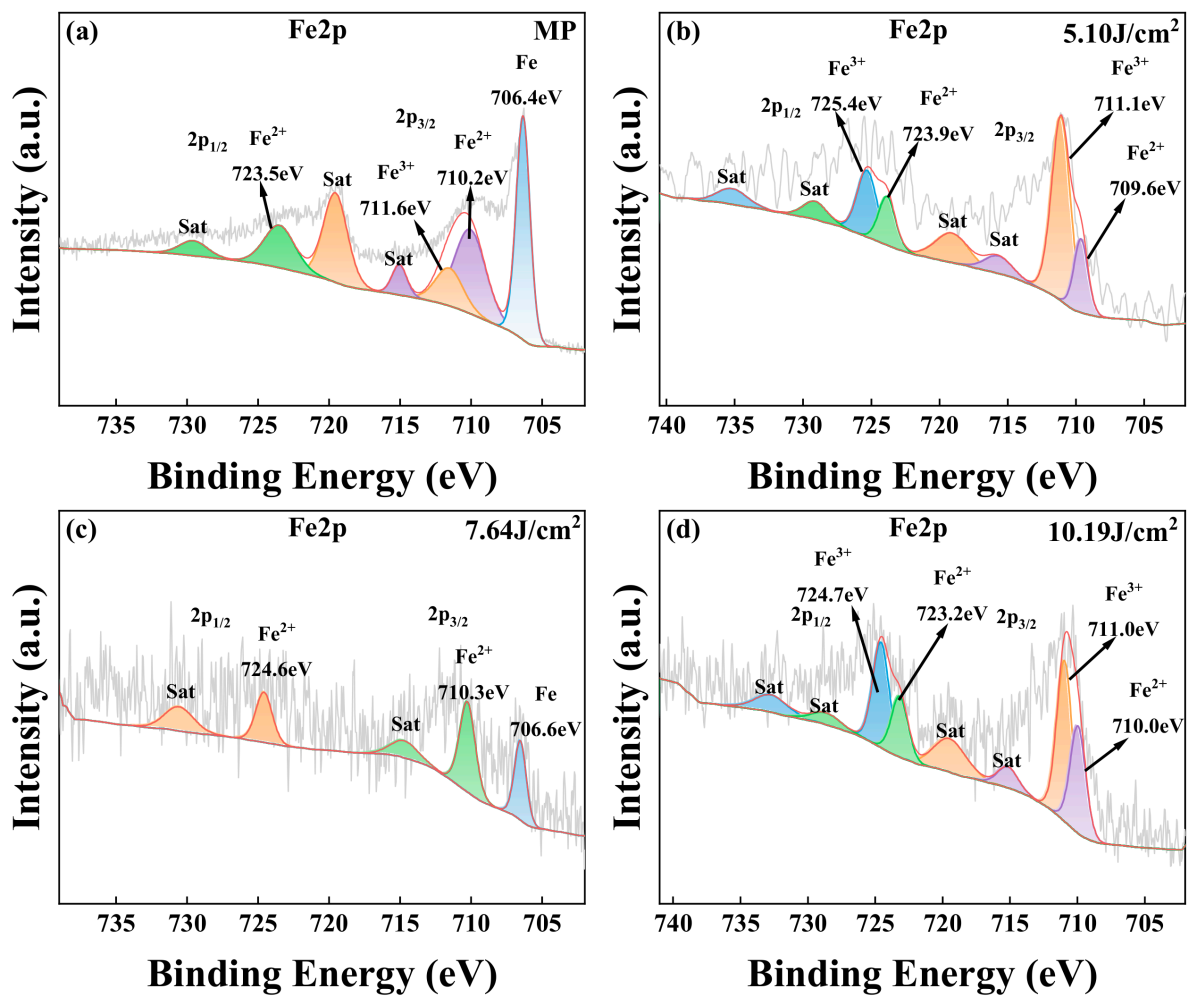
Fe 2p spectra of the specimens before and after laser cleaning. (**a**) Spectral data for the polished specimen; (**b**) Spectral data for the specimen at 5.10 J/cm^2^; (**c**) Spectral data for the specimen at 7.64 J/cm^2^; (**d**) Spectral data for the specimen at 10.19 J/cm^2^.

The surface composition of the mechanically ground and laser-cleaned specimens (argon atmosphere) was analyzed. Figure 7 shows the Fe 2p spectra of the sample surface. The brown-black surface oxide/rust layer on the original specimen was mainly composed of iron oxides, including hematite (Fe_2_O_3_) and magnetite (Fe_3_O_4_), together with iron oxyhydroxides such as α-FeOOH and γ-FeOOH [31,32]. Rust formation generally occurs under two conditions: high humidity with low oxygen content, or through oxidation of atmospheric Fe_3_O_4_, as described by Equations (4) and (5) [31]. As shown in Figure 7a, the original sample surface contained a rust layer, so Fe^2+^ and Fe^3+^ peaks were present. Combined with the SEM morphology in Figure 5a, various impurities adhered to the surface and hindered acquisition of the Fe 2p signal, resulting in a relatively small Fe 2p peak area. In Figure 7b, after cleaning at 5.10 J/cm^2^, the Fe^2+^ and Fe^3+^ peaks were clearly visible, indicating that the low fluence could only partially remove the oxide film and that considerable oxidation products still remained on the surface. In Figure 7c, after cleaning at 7.64 J/cm^2^, the Fe^3+^ peak disappeared, the Fe^2+^ peak area decreased, and an Fe^0^ peak appeared. At 7.64 J/cm^2^, a contribution near the binding-energy region associated with metallic Fe became detectable, whereas the Fe^3+^-related contribution was no longer clearly resolved. Together with the minimum oxygen content, the O 1s spectrum, and the surface morphology, these results are consistent with effective removal of the oxidized surface layer and increased exposure of the metallic substrate. In Figure 7d, after cleaning at 10.19 J/cm^2^, the Fe^3+^ peak reappeared with a larger area, and the Fe^2+^ peak was still present, suggesting the formation of iron-containing oxides again, which explains the brown traces on the surface (Figure 4).

(4) $$Fe_{2}O_{3}+H_{2}O⇌2\mathrm{FeOOH}$$

(5) $$4{\mathrm{Fe}}_{3}O_{4}+O_{2}\to 6{\mathrm{Fe}}_{2}O_{3}$$

**Figure 8:**
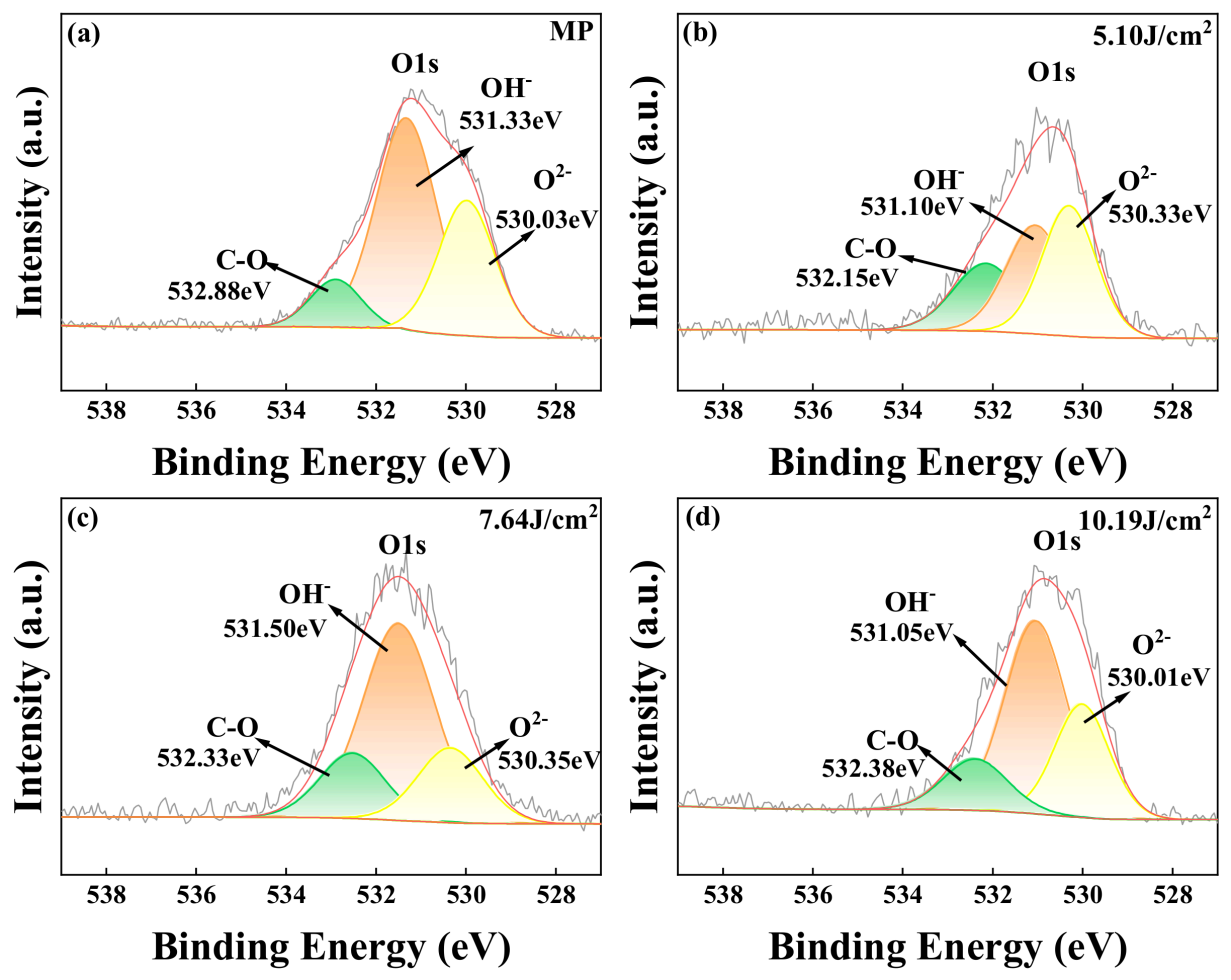
The O 1s spectra of the samples before and after laser cleaning. (**a**) Spectral data for the polished specimen; (**b**) Spectral data for the specimen at 5.10 J/cm^2^; (**c**) Spectral data for the specimen at 7.64 J/cm^2^; (**d**) Spectral data for the specimen at 10.19 J/cm^2^.

Figure 8 shows the changes in the O 1s spectral peaks of the above samples. In Figure 8a, the spectrum of the ground sample surface shows a relatively small O^2−^ peak area because of impurities, loose rust, and other contaminants. In Figure 8b, during cleaning at 5.10 J/cm^2^, the laser first removed the loose surface contaminants and part of the oxidation products, exposing underlying oxides that had not yet been completely removed. As a result, the detectability of surface oxygen species increased and the O^2−^ peak area actually became larger. In Figure 8c, after cleaning at 7.64 J/cm^2^, the oxide-related surface signal was substantially reduced, and no obvious secondary oxidation was detected in the analyzed area; the surface O content was the lowest of all samples (Figure 6), and thus the O^2−^ peak area decreased. In Figure 8d, after cleaning at 10.19 J/cm^2^, the O^2−^ peak area increased. This suggests that the high laser energy caused a sharp rise in surface temperature and secondary oxidation, leading to increased O content (Figure 6). The enlarged Fe^3+^ peak also indicates the formation of new iron oxides. Therefore, the coupled evolution of the Fe 2p and O 1s spectra not only reflects the extent of oxide-layer removal but also reveals the competition between removal and reoxidation during laser cleaning.

The XPS results show that, with increasing laser fluence, the sample surface progressively changes from being dominated by Fe^3+^ oxidation states to exposing Fe^0^, while slight reoxidation appears under high-energy conditions. This indicates that argon-assisted laser cleaning can effectively remove the oxide layer while suppressing secondary oxidation, but excessive energy input still weakens the surface-cleaning effect because of local thermal accumulation.

Because the Fe 2p spectra contain complex multiplet and satellite structures, the chemical-state assignments are interpreted qualitatively rather than as definitive quantitative phase identification. Therefore, the conclusion regarding oxide removal is based on the combined evidence from the Fe 2p and O 1s spectra, EDS oxygen content, and surface morphology.

#### 3.1.4. Three-Dimensional Morphology and Surface Roughness Analysis

**Figure 9:**
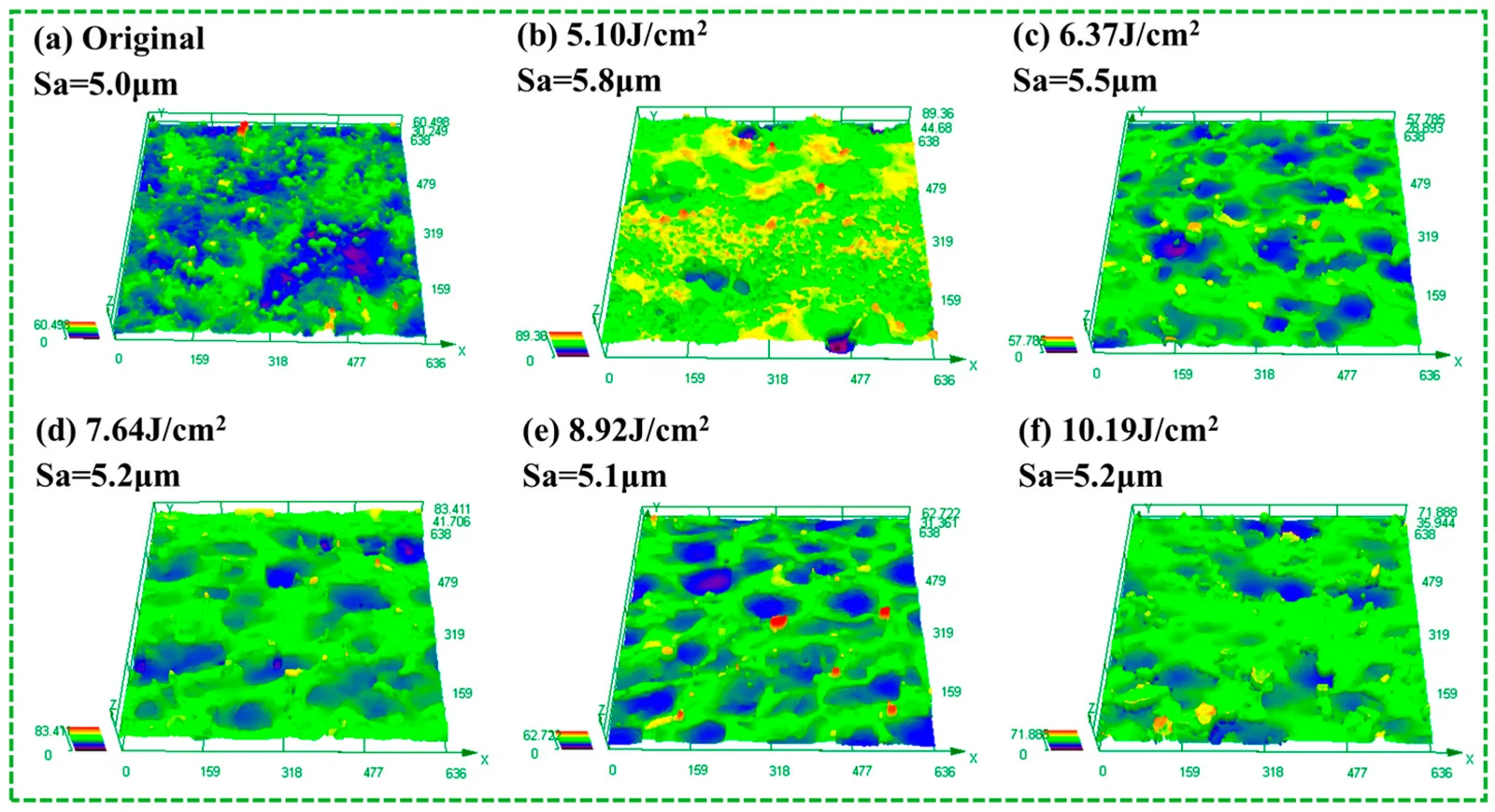
Three-dimensional surface morphology of the original and laser-cleaned specimens.

**Figure 10:**
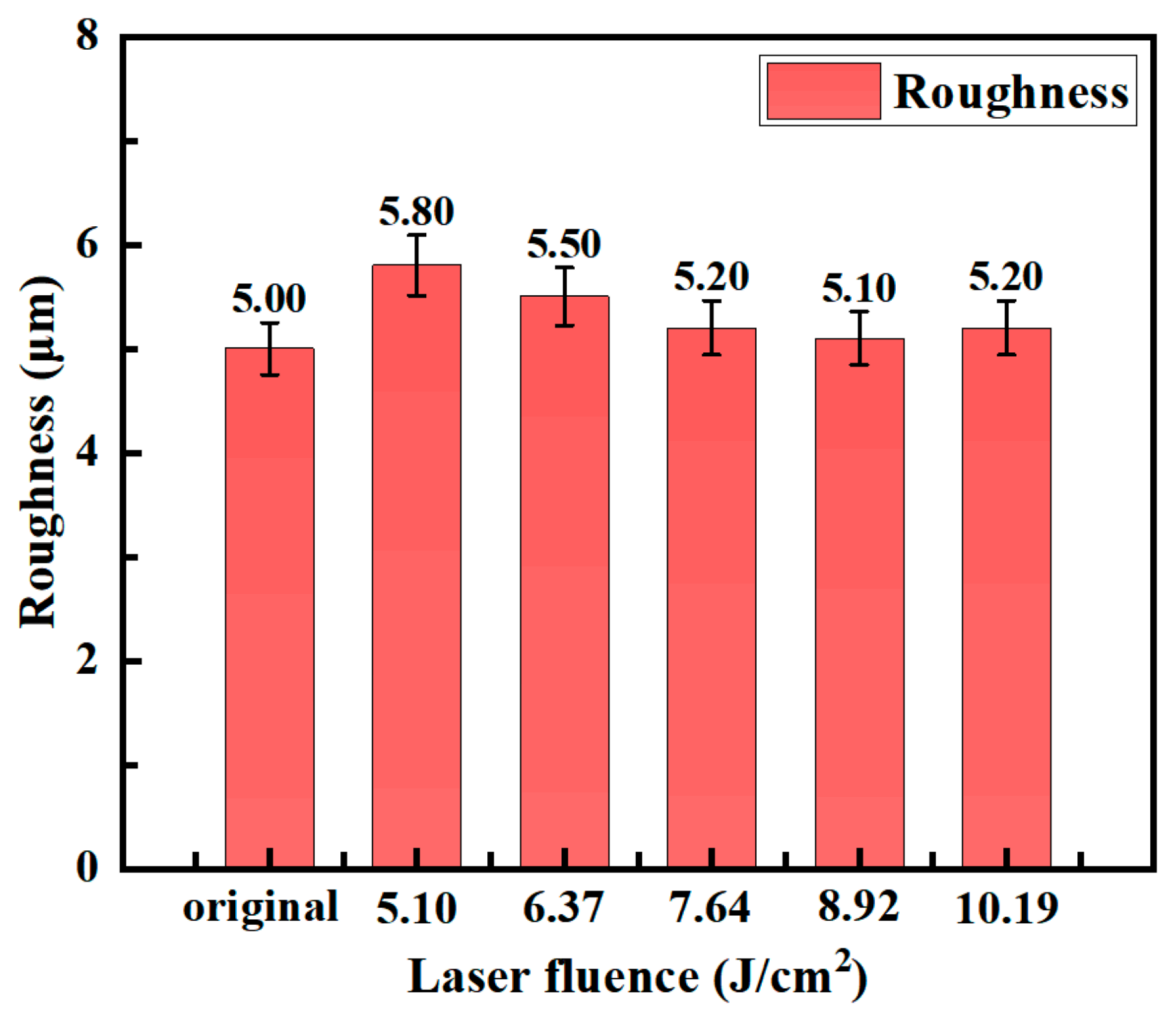
Surface roughness of the original and laser-cleaned specimens.

Figure 9 and Figure 10 show the three-dimensional surface morphology and roughness evolution of the original and laser-cleaned specimens. The surface roughness Sa exhibits a trend of first increasing and then decreasing. As shown in Figure 9a, the original specimen surface contains fragmented particles and irregular pits, mainly associated with uneven rust accumulation, spallation, and corrosion pits. Because some pits were filled by contaminants attached to the surface, the height difference on the original surface was small and the Sa value was 5.0 μm.

At a fluence of 5.10 J/cm^2^, the surface relief became much more pronounced and Sa reached its maximum value of 5.8 μm. As indicated by the 3D morphology in Figure 9b, many ridge-and-valley protrusions were present on the specimen surface, showing that rust-layer removal was uneven at low fluence. In some areas the rust peeled and flaked off, while in others rust residues or solidified structures remained, increasing the surface height difference and thus the roughness.

As the laser fluence increased to 6.37 J/cm^2^, the surface peaks and valleys became less pronounced and Sa decreased to 5.5 μm. When the fluence further increased to 7.64–10.19 J/cm^2^, the surface became progressively flatter, and Sa values were 5.2 μm, 5.1 μm, and 5.2 μm, respectively. As shown in Figure 9d–f, increasing laser energy improved the cleaning of the rust layer, so protrusions formed by rust-product accumulation gradually decreased. Meanwhile, the higher surface temperature increased melting, and the molten metal flowed and spread under surface tension, filling low-lying regions. The combined reduction in protrusions and pits lowered the overall surface relief. This is consistent with the micro-morphology in Figure 5, which evolves gradually from crater-like features to a flatter remelted morphology.

#### 3.1.5. Analysis of Surface-Layer Microstructure and Strain Characteristics Before and After Laser Cleaning

**Figure 11:**
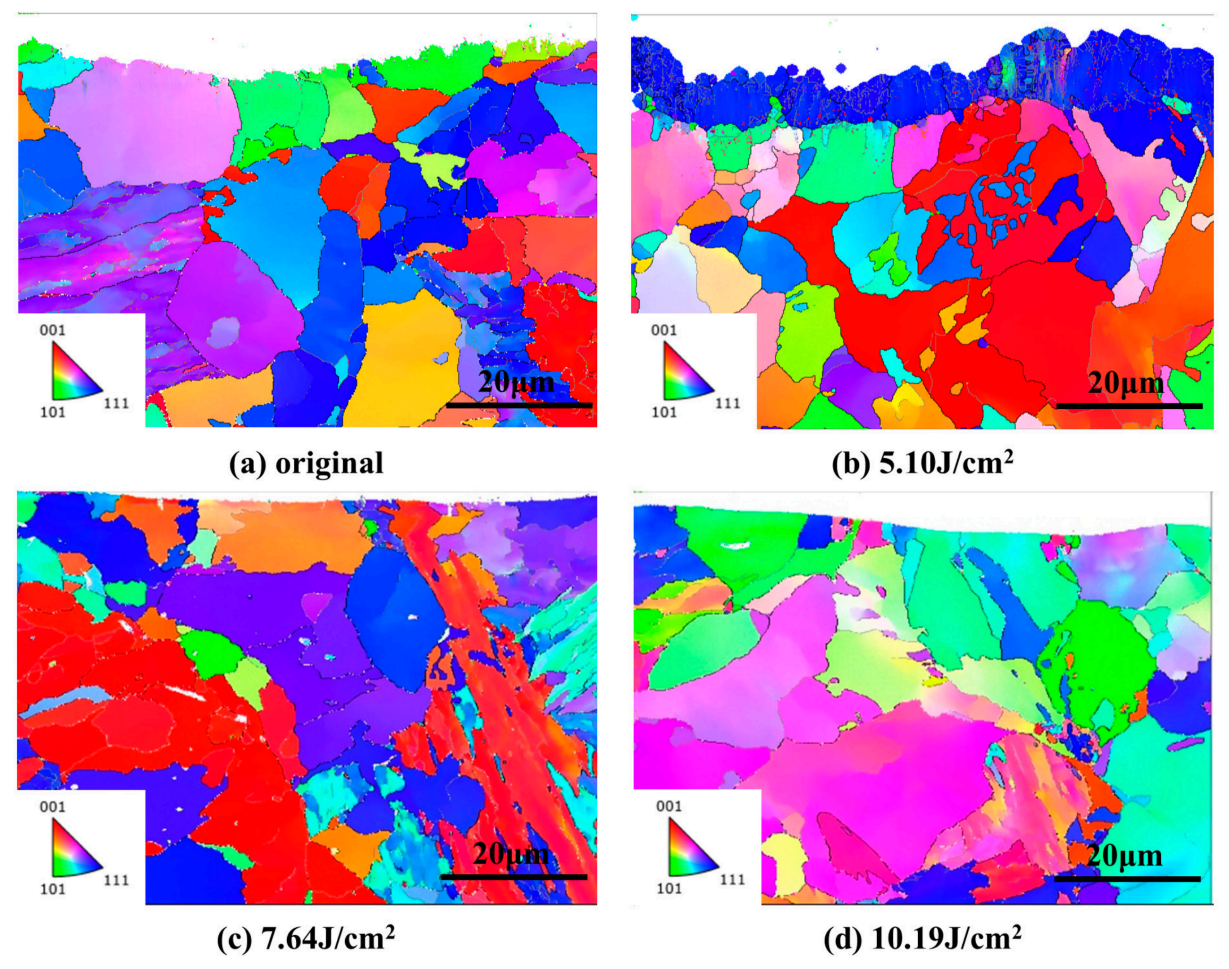
EBSD maps of the specimen cross-sections before and after laser cleaning. The analyzed area for each map was 57.5 μm × 42 μm. The numbers of analyzed grains in panels (**a**–**d**) were 2436, 5607, 2316, and 1439, respectively.

Figure 11 shows the EBSD maps of the cross-sections of the original and laser-cleaned specimens. The analyzed EBSD regions measured 57.5 μm × 42 μm. After EBSD data acquisition and processing, the numbers of analyzed grains in panels (a–d) were 2436, 5607, 2316, and 1439, respectively. The corresponding average grain sizes were 10.23, 13.75, 12.38, and 18.86 μm, respectively. The grain-orientation distribution and grain morphology remained broadly similar among the examined conditions, and no obvious systematic change in the overall grain structure was observed within the analyzed regions. These results indicate that, within the examined cross-sectional area and the resolution of the EBSD measurements, the laser treatment mainly affected the near-surface region and did not produce obvious changes in the measured internal grain structure.

**Figure 12:**
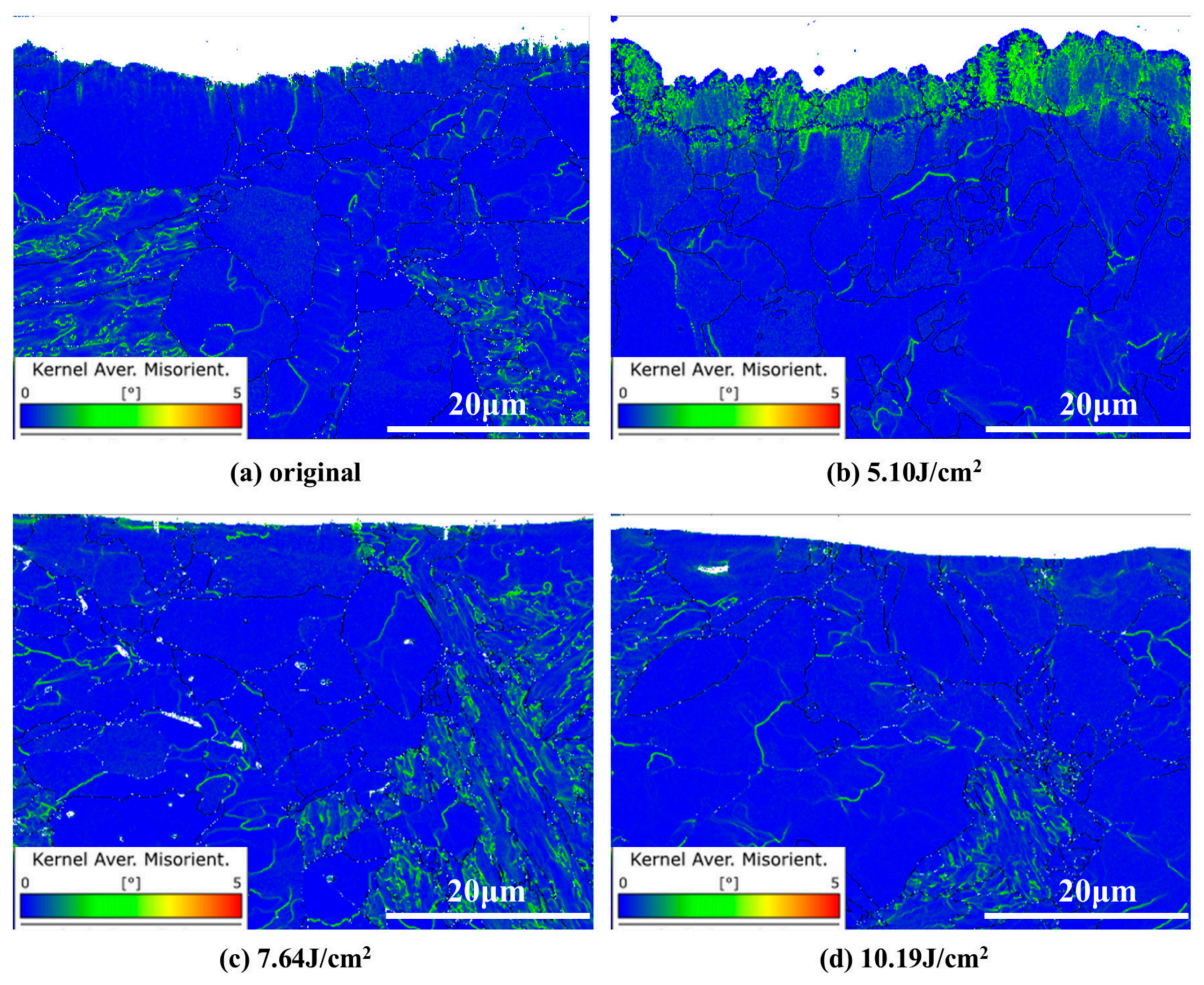
KAM maps of the specimen cross-sections before and after cleaning.

Figure 12 shows the KAM (kernel average misorientation) maps of the cross-sections of the original and laser-cleaned specimens. The KAM value reflects local orientation differences and lattice distortion within grains and is therefore commonly used to characterize local strain concentration and dislocation-density variations. Blue regions correspond to lower KAM values, indicating smaller lattice-orientation differences; green lines indicate higher KAM values, suggesting greater strain concentration and higher dislocation density.

In Figure 12a, the green regions in the near-surface layer of the original specimen indicate relatively high local misorientation and pronounced orientation gradients, whereas the interior of the substrate shows lower KAM values. At a fluence of 5.10 J/cm^2^, a distinct high-KAM region remains near the surface, indicating that local orientation gradients and strain heterogeneity are still pronounced. Combined with the XPS results and SEM morphology, this observation is consistent with the presence of residual surface products and solidification features. When the fluence increases to 7.64–10.19 J/cm^2^, the KAM values in the near-surface layer decrease, indicating reduced local misorientation and weaker orientation gradients in the examined regions. This change may be associated with the reduction of heterogeneous surface products and the rapid heating and cooling induced by laser irradiation. However, stress relaxation or a specific change in dislocation density cannot be conclusively established from the KAM data alone.

### 3.2. Cleaning Mechanism Study

**Figure 13:**
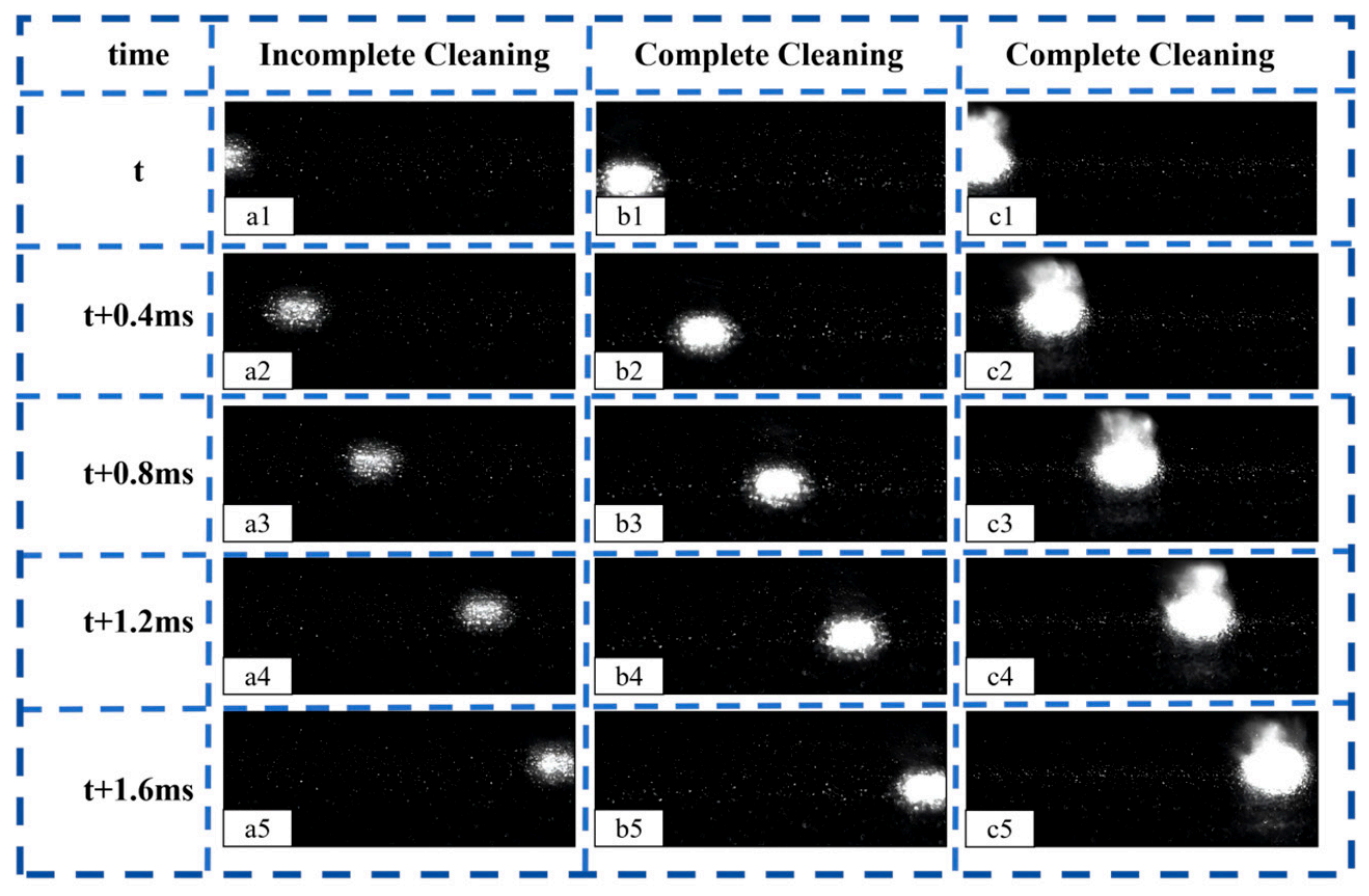
Dynamic behavior of the sample surface during cleaning in the two atmospheres—(**a1**–**a5**): 5.10 J/cm^2^; (**b1**–**b5**): 7.64 J/cm^2^; (**c1**–**c5**): 10.19 J/cm^2^.

To clarify the oxide-removal mechanism during nanosecond laser cleaning, the results of surface morphology, elemental composition, chemical-state analysis, and high-speed imaging were considered together. Based on the combined characterization results, three representative fluences were selected for detailed analysis: 5.10 J/cm^2^, representing incomplete cleaning; 7.64 J/cm^2^, representing effective cleaning; and 10.19 J/cm^2^, representing over-cleaning. The corresponding dynamic behaviors are shown in Figure 13.

In the high-speed images, the laser-irradiated region appears as the brightest region. The region above the bright spot represents the surface that has not yet been cleaned, whereas the region below it represents the surface that has already been scanned. As the laser fluence increased from 5.10 to 10.19 J/cm^2^, the interaction width increased from approximately 1.2 to 2.5 mm, and the plasma plume became progressively more intense. This indicates that increasing the fluence enhanced the energy deposition and intensified the interaction between the laser beam, the oxide layer, and the substrate.

At 5.10 J/cm^2^, the surface morphology and oxygen-content results indicate that the oxide layer was not completely removed. The high-speed images show a relatively weak plasma plume and limited particle ejection. Under this condition, the absorbed laser energy was insufficient to generate intensive ablation of the oxide layer. The removal process was therefore mainly associated with local heating, limited particle detachment, and a weak plasma-shock effect. Because a considerable amount of oxide remained on the surface, the cleaning was incomplete.

When the fluence increased to 7.64 J/cm^2^, the plasma plume became more pronounced and particle ejection was enhanced. The XPS results showed the disappearance of the Fe^3+^ signal and the appearance of the Fe^0^ signal, while the oxygen content reached its minimum value of 2.11 wt.%. These results confirm that the oxide film was effectively removed and that the metallic substrate was exposed. At this stage, the absorbed energy was sufficient to vaporize and ablate the oxide layer, while the expansion of the laser-induced plasma generated a shock effect that assisted particle detachment. Therefore, effective cleaning at 7.64 J/cm^2^ resulted from the combined action of laser ablation, plasma shock, and particle detachment.

At 10.19 J/cm^2^, the interaction region became wider, approximately 2.5 mm, and the plasma plume and particle ejection were the most intense. The increased energy input promoted stronger ablation and more vigorous material ejection. However, the surface oxygen content increased again, and the Fe^3+^ signal reappeared in the XPS spectrum. These results indicate that the excessive thermal input caused local melting or remelting of the substrate and promoted secondary oxidation after the original oxide layer had been removed. Thus, although ablation remained the dominant removal mechanism at this fluence, the cleaning process was accompanied by an increased risk of substrate damage and reoxidation.

**Figure 14:**
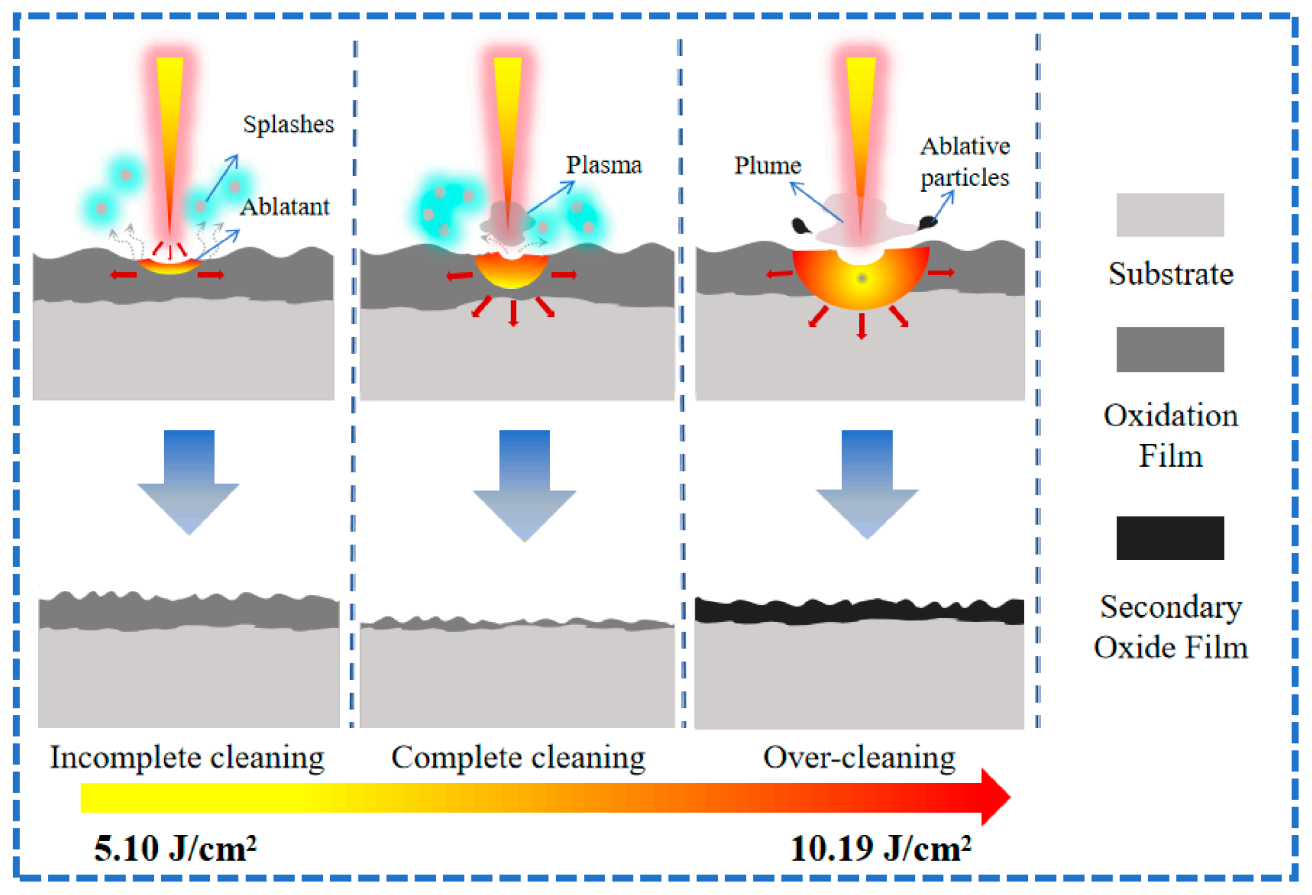
Mechanism of oxide-layer removal from the surface of 10CrNi2Mo3Cu2V steel.

Laser cleaning involves the coupling of optical, thermal, and mechanical effects [33]. Depending on the laser fluence, the dominant removal behavior changes from insufficient particle detachment at low fluence, to the synergistic action of plasma shock and laser ablation at intermediate fluence, and finally to intense ablation accompanied by remelting and secondary oxidation at excessive fluence. The corresponding removal process is schematically illustrated in Figure 14.

At low fluence, the energy input is mainly consumed by heating the oxide layer, and the oxide film cannot be completely removed. At an appropriate fluence, the oxide layer absorbs sufficient energy to undergo rapid melting, vaporization, and ejection, while the laser-induced plasma assists the detachment of oxide particles from the surface. At excessive fluence, the higher thermal input increases the likelihood of substrate melting and secondary oxidation. Therefore, effective laser cleaning requires a balance between sufficient energy for oxide-film removal and limited thermal input to the substrate.

### 3.3. Post-Cleaning Performance

#### 3.3.1. Surface Microhardness After Laser Cleaning

**Figure 15:**
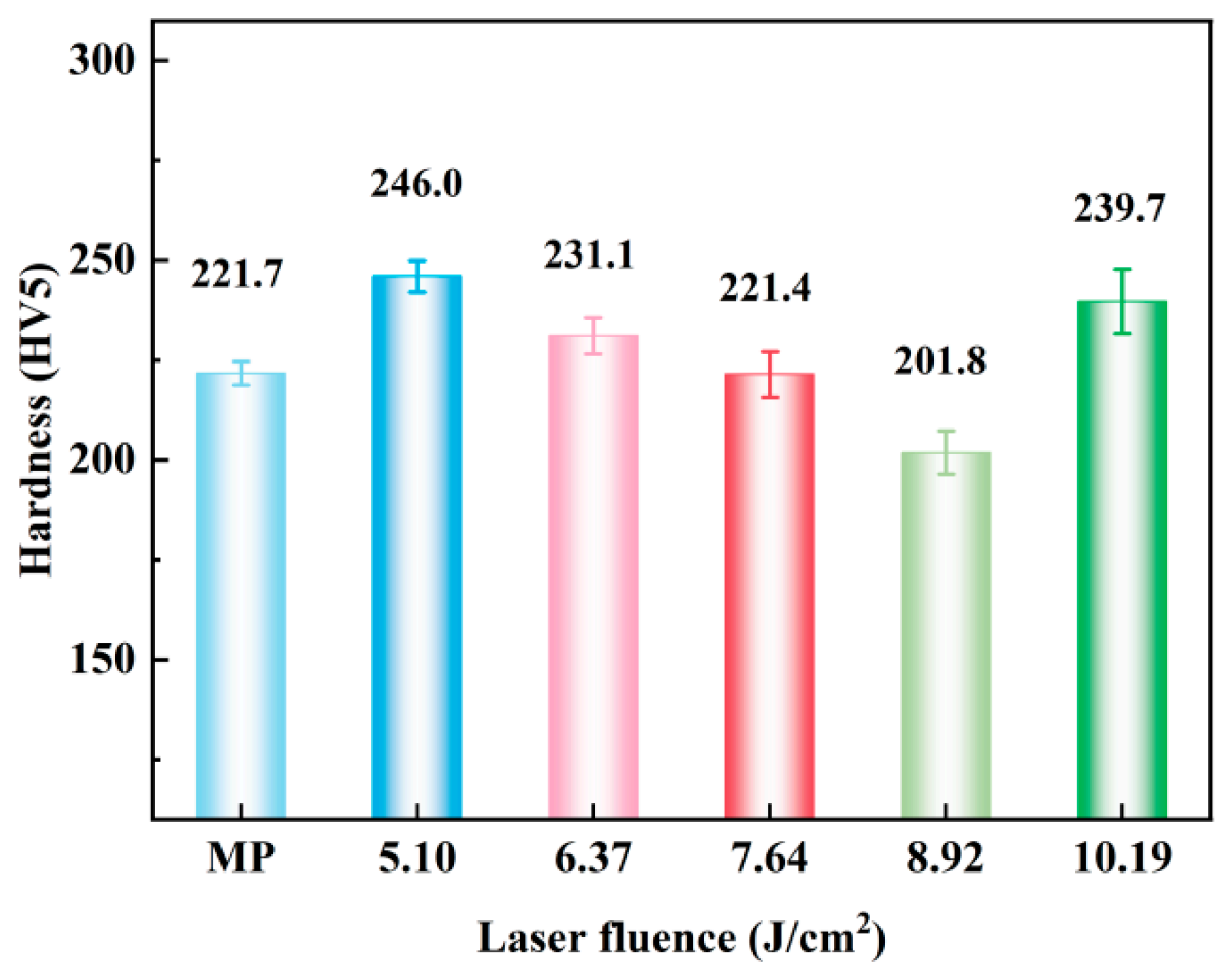
Surface microhardness of the mechanically ground specimen and the laser-cleaned specimens at different fluences.

Figure 15 shows the surface microhardness of the mechanically ground (MP) specimen and the laser-cleaned specimens at different fluences. A check of the data shows that hardness first increases, then decreases, and then increases again with increasing fluence: MP, 221.7 HV5; 5.10 J/cm^2^, 246.0 HV5; 6.37 J/cm^2^, 231.1 HV5; 7.64 J/cm^2^, 221.4 HV5; 8.92 J/cm^2^, 201.8 HV5; and 10.19 J/cm^2^, 239.7 HV5.

At 5.10 J/cm^2^, the surface hardness is highest, mainly not because of substrate strengthening but because a relatively large amount of oxide and ablation products remain on the surface. Combined with the EBSD results above, the surface layer at this condition is still affected by laser heating, with high local dislocation density and KAM values. The heterogeneous residual layer and the remelted structure jointly increase the indentation response.

When the fluence increases to 6.37–7.64 J/cm^2^, the rust layer is removed more completely, and the EDS/XPS results show that the surface oxygen content continues to decrease, reaching a low level at 7.64 J/cm^2^. At the same time, EBSD indicates that cleaning mainly affects the surface layer and that the grain orientation and size in the substrate remain essentially unchanged. This suggests that this fluence range is dominated by reduced surface residue and structural stability rather than bulk strengthening.

With further increase to 8.92 J/cm^2^, the hardness decreases to 201.8 HV5, indicating that this surface state is most favorable for reducing the indentation response, possibly because the rust layer has been removed more thoroughly while secondary oxidation has not yet become pronounced. This trend cannot be explained by EBSD alone; a more appropriate interpretation combines EDS/XPS with surface morphology. At 8.92 J/cm^2^, surface oxides and ablation residues are further reduced, but there is still no obvious secondary oxidation or remelting such as that seen at 10.19 J/cm^2^, so the hardness first drops to its minimum and then increases as the energy becomes too high.

When the fluence further increases to 10.19 J/cm^2^, the hardness rises again to 239.7 HV5. Combined with the XPS results, the O content on the surface increases again and all characteristic peaks are strengthened, indicating that the high energy input may cause secondary oxidation or remelting of the surface. Meanwhile, the KAM results show that the local misorientation in the surface layer increases under high-energy conditions, implying higher dislocation density and local strain. Therefore, the hardness recovery at this condition is mainly attributed to high-temperature-induced secondary oxidation or remelting, together with increased local strain.

#### 3.3.2. Weld Microstructure Observation

**Figure 16:**
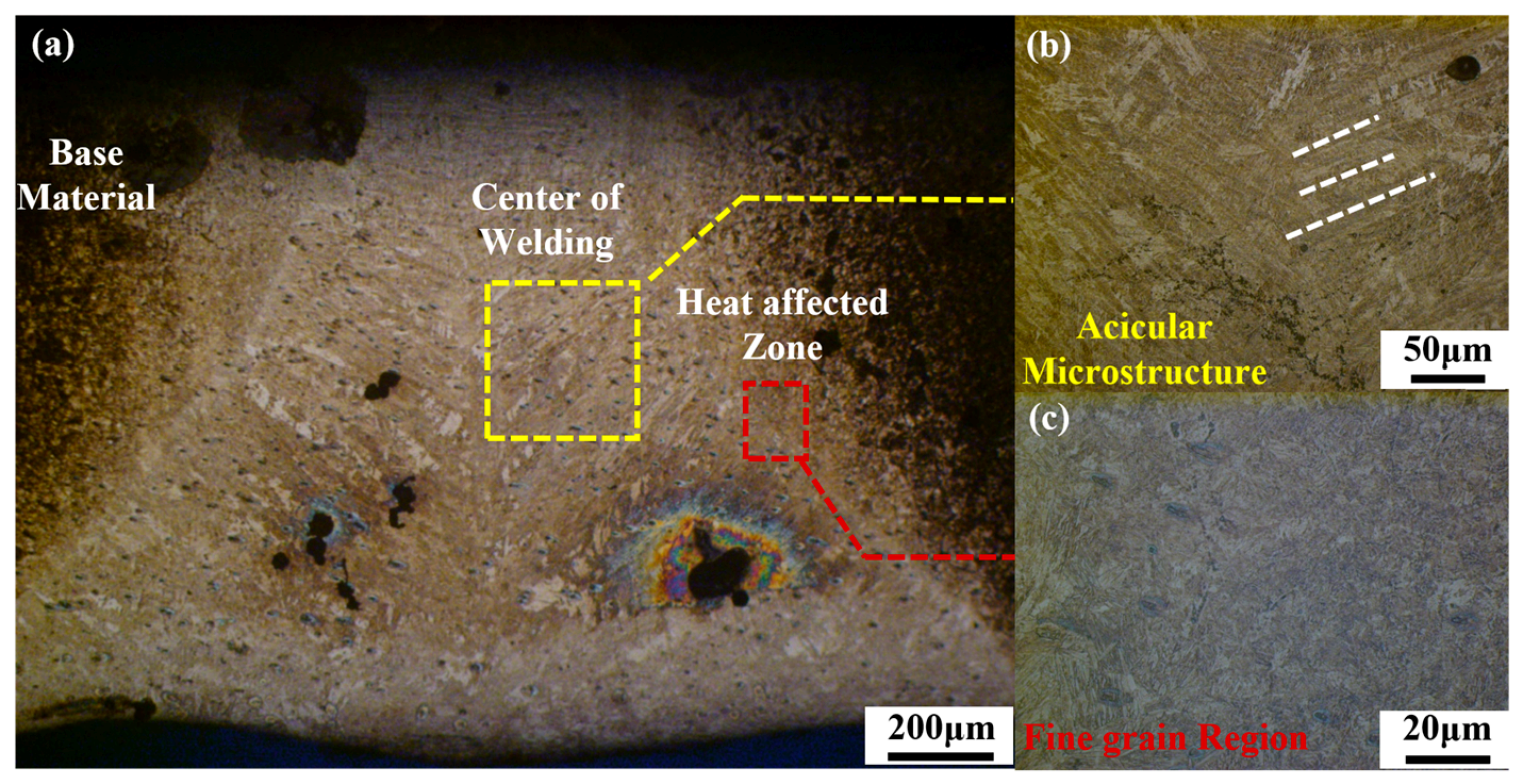
Weld microstructure: (**a**) complete weld region; (**b**) center zone; (**c**) heat-affected zone.

Under the optimal cleaning conditions (argon protection, fluence 7.64 J/cm^2^), the weld microstructure of the cleaned 10CrNi2Mo3Cu2V steel joint was observed. Figure 16 shows that the cross-section of the joint mainly consists of the base metal, weld center, and heat-affected zone, with distinct microstructural differences among these regions. During welding, the molten pool undergoes repeated melting and solidification, so the weld microstructure exhibits a certain degree of directionality. As shown in Figure 16b, the weld center is dominated by acicular martensite arranged in an interwoven manner, which can reduce stress concentration to some extent and increase weld strength. The heat-affected zone in Figure 16c has finer grains and less obvious texture. Although this region does not fully melt, it becomes austenitized under thermal cycling and forms a fine, uniform microstructure after rapid cooling, which is beneficial to the structural stability of the heat-affected zone.

**Figure 17:**
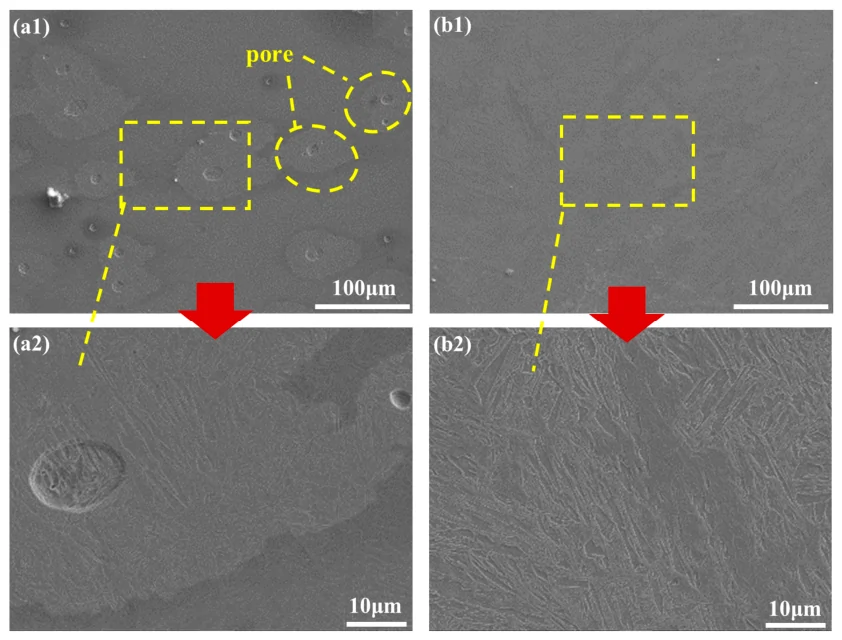
SEM morphology of the weld cross-section: (**a1**,**a2**) mechanical grinding; (**b1**,**b2**) 7.64 J/cm^2^.

Figure 17 shows the SEM morphology of the weld center under different treatment conditions. As shown in Figure 17(a1), the mechanically ground specimen weld contains many circular pores, and some pores are relatively large. Further magnification in Figure 17(a2) reveals acicular structures near the pores. The pores disrupt the continuity of the weld metal and may reduce joint quality. This indicates that although mechanical grinding can remove most rust, grinding debris and contaminants may still remain on the surface and participate in melt-pool reactions during welding, affecting gas escape and thereby increasing the probability of pore formation.

For the specimen cleaned with the optimal parameters, no obvious pores were observed in the weld center, and the acicular structures appeared more continuous and intact at higher magnification. Combined with the surface O content and XPS results above, this indicates that rust and contaminants were effectively removed after cleaning, reducing the sources of inclusions and gas during welding and thereby significantly suppressing pore defects.

Overall, compared with mechanical grinding, laser cleaning offers a more pronounced advantage in improving the preweld surface state. It not only removes surface contaminants but also reduces the tendency for pore formation during welding, thereby helping to improve joint quality.

#### 3.3.3. Effect of Cleaning on the Tensile Properties of the Joint

**Figure 18:**
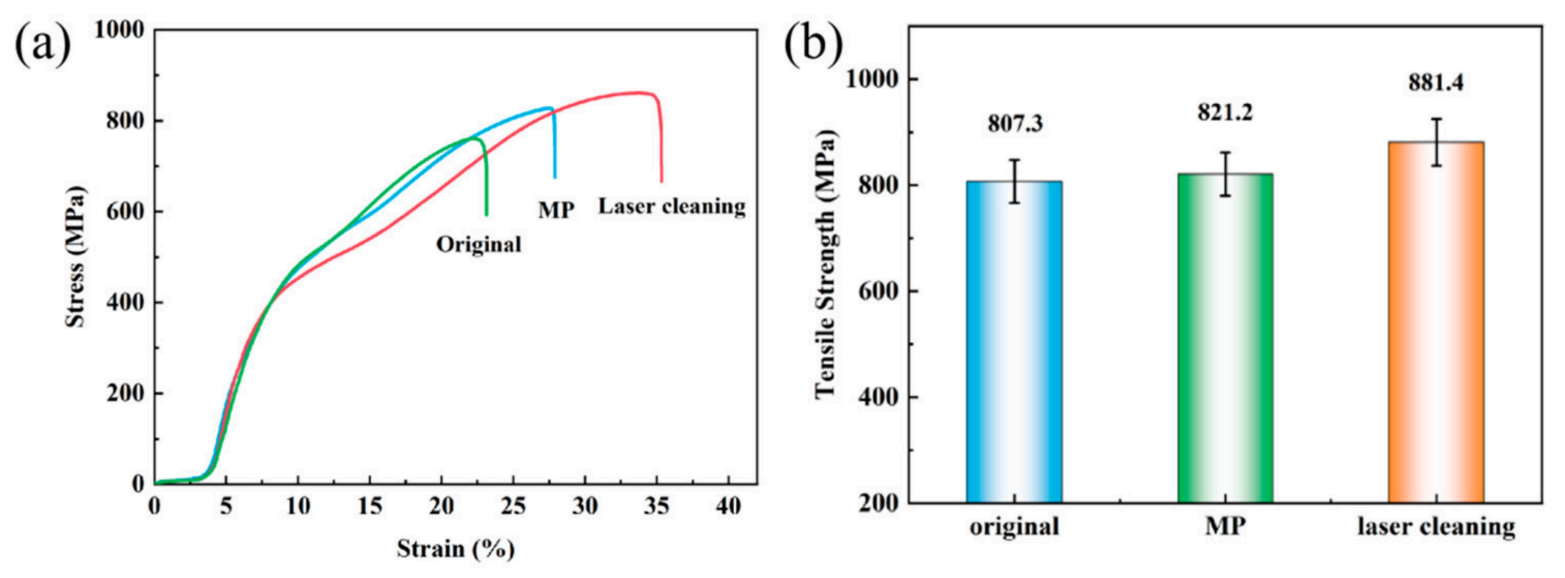
(**a**) Tensile curves of the welded joints at room temperature; (**b**) tensile strength of the welded specimens after different treatments. The data are presented as the mean ± standard deviation of three specimens for each condition.

To evaluate the influence of different preweld surface treatments on the mechanical properties of alloy-steel welded joints, the original specimen, the mechanically ground (MP) specimen, and the specimen cleaned with the optimal process parameter (7.64 J/cm^2^) were welded, and longitudinal tensile tests were performed on the welded joints to obtain the tensile properties of the welds. Figure 18a shows the stress–strain curves of the three specimens, and Figure 18b compares the tensile strengths of the three sets of parallel specimens.

As shown in Figure 18a, the stress–strain curves of all three specimens pass through elastic deformation, plastic deformation, and fracture. However, the laser-cleaned welded specimen can sustain a higher stress level during plastic deformation, and its elongation is significantly higher than that of the original welded specimen and the MP welded specimen, at 35.3, 23.1, and 27.9, respectively. This indicates that laser cleaning can significantly improve the ductility and toughness of the joint.

As shown in Figure 18b, the mean tensile strengths of the untreated, mechanically ground, and laser-cleaned welded specimens were 807.3 ± 8.31 MPa, 821.2 ± 5.60 MPa, and 881.4 ± 9.85 MPa, respectively (*n* = 3). Mechanical grinding increased the mean tensile strength by 13.9 MPa compared with the untreated condition, indicating that it partially improved the pre-weld surface condition. Laser cleaning produced a more pronounced improvement, increasing the mean tensile strength by 60.2 MPa compared with mechanical grinding and by 74.1 MPa compared with the untreated condition. In addition, the laser-cleaned joints exhibited a higher elongation of 35.3%, compared with 23.1% for the untreated joints and 27.9% for the mechanically ground joints. These results indicate that optimized laser cleaning can simultaneously improve the strength and ductility of the welded joints.

Combined with the SEM morphology of the weld cross-section, the MP welded specimen contains many pore defects, whereas the laser-cleaned specimen shows no obvious pores, which is closely related to the preweld surface state. After grinding, the specimen surface may still retain rust, debris, and other contaminants, leading to a higher O content and making the surface a potential gas source during welding, thereby increasing the tendency for pore formation. In contrast, laser cleaning more thoroughly removes the rust layer and raises the surface cleanliness to nearly Sa3 level, reducing the formation of pore defects. Overall, laser cleaning under suitable parameters can effectively improve the preweld surface condition of 10CrNi2Mo3Cu2V alloy steel and thereby enhance the strength and ductility of the welded joint.

In this study, the optimal fluence was selected using a hierarchical engineering criterion. The primary requirement was effective removal of the surface oxide/rust layer while avoiding obvious secondary oxidation. This criterion was satisfied at 7.64 J/cm^2^, where the surface oxygen content reached its minimum value of 2.11 wt.%, the Fe^3+^-related contribution was no longer clearly resolved, and the metallic-Fe contribution became detectable in the Fe 2p spectrum. The bright metallic appearance of the surface under argon protection further supported the absence of obvious secondary oxidation.

Surface integrity and subsequent welding performance were then used to validate the selected condition. At 7.64 J/cm^2^, the surface roughness was 5.2 μm and the microhardness was 221.4 HV5, which was comparable to that of the mechanically ground specimen. The corresponding welded joint was pore-free and exhibited a mean tensile strength of 881.4 MPa. Although the roughness at 8.92 J/cm^2^ was slightly lower, the oxygen content increased to 2.27 wt.%, indicating that secondary oxidation had begun. Therefore, 7.64 J/cm^2^ was considered the optimum condition because it provided the best balance between oxide removal, oxidation control, surface integrity, and welding performance.

## 4. Conclusions

This study investigated the nanosecond laser-cleaning process for the oxide film on 10CrNi2Mo3Cu2V alloy steel. The cleaning effectiveness was verified, the removal mechanism was clarified, and the influence of laser cleaning on vacuum electron-beam welding quality was analyzed. The main conclusions are as follows:

(1) Nanosecond laser cleaning under argon protection can effectively remove the oxide film on 10CrNi2Mo3Cu2V alloy steel. As fluence increases, the surface oxygen content first decreases and then increases, reaching a minimum of 2.11 wt.% at 7.64 J/cm^2^. The XPS results provide chemical-state evidence consistent with effective removal of the oxidized surface layer, and argon suppresses secondary oxidation. Under the optimal conditions, the substrate surface is uniform and the roughness decreases to 5.2 μm.
(2) Under the investigated conditions, laser cleaning had only a limited effect on the measured surface hardness, and no obvious thermal damage was detected in the examined areas using the applied characterization methods. The tensile strength of the welded joint after pretreatment under the optimal parameters reaches 881.4 MPa, and the ductility and toughness are improved, showing that preweld laser cleaning can optimize the mechanical properties of the joint.
(3) Oxide-film removal relies on the synergistic action of laser ablation and plasma shock. At low energy, oxide removal is incomplete; at high energy, surface remelting and secondary oxidation are easily induced. The cleaning process is therefore a competition between effective film removal and thermal damage/reoxidation.
(4) Based on the hierarchical criterion of effective oxide-layer removal without obvious secondary oxidation, together with acceptable surface integrity and improved welding performance, the process window was determined to be 6.37–7.64 J/cm^2^. Within this window, 7.64 J/cm^2^ was selected as the optimal fluence because it produced the minimum oxygen content, favorable Fe chemical-state characteristics, a surface roughness of 5.2 μm, hardness comparable to that of the ground specimen, and a pore-free welded joint with the highest mean tensile strength.

## Figures and Tables

**Table 1:** Chemical composition of 10CrNi2Mo3Cu2V steel.

**Element**	**Cr**	**Ni**	**Mo**	**Cu**	**V**	**C**	**Fe**
**Content (%)**	**0.8~1.2**	**1.8~2.2**	**2.8~3.2**	**1.8~2.2**	**0.1~0.2**	**0.08~0.12**	**Balance**

**Table 2:** Laser-cleaning process parameters.

Parameter	Value
Wavelength	1064 nm
Pulse width	100 ns
Spot diameter (*D*)	1 mm
Laser power (*P*)	400 W, 500 W, 600 W, 700 W, 800 W
Laser fluence (*F*)	5.10 J/cm^2^, 6.37 J/cm^2^, 7.64 J/cm^2^, 8.92 J/cm^2^, 10.19 J/cm^2^
Frequency (*f*)	10 kHz
Scanning speed (*v*)	5000 mm/s
Transverse overlap ratio (*U_x_*)	50%
Longitudinal overlap ratio (*U_y_*)	90%
Cleaning times	1
Displacement perpendicular to scanning direction (*L*)	100 μm
Environment	Air/Argon
Argon flow rate	10 L/min
Argon purity	99.999%

## Data Availability

The original contributions presented in this study are included in the article. Further inquiries can be directed to the corresponding authors.
